# Development and Diversity of Epibiont Assemblages on Cultivated Sugar Kelp (*Saccharina latissima*) in Relation to Farming Schedules and Harvesting Techniques

**DOI:** 10.3390/life13010209

**Published:** 2023-01-11

**Authors:** Sophie Corrigan, A. Ross Brown, Charles R. Tyler, Catherine Wilding, Carly Daniels, Ian G. C. Ashton, Dan A. Smale

**Affiliations:** 1 Faculty of Health and Life Sciences, University of Exeter, Geoffrey Pope Building, Stocker Road, Exeter EX4 4QD, UK; 2 Sustainable Aquaculture Futures, University of Exeter, Exeter EX4 4QD, UK; 3 Marine Biological Association of the United Kingdom, The Laboratory, Citadel Hill, Plymouth PL1 2PB, UK; 4 College of Engineering, Mathematics and Physical Sciences, University of Exeter, Penryn Campus, Cornwall TR10 9FE, UK

**Keywords:** seaweed aquaculture, biofouling, partial harvesting, ecosystem approach to aquaculture, macroalgal cultivation

## Abstract

Seaweed farming in Europe is growing and may provide environmental benefits, including habitat provisioning, coastal protection, and bioremediation. Habitat provisioning by seaweed farms remains largely unquantified, with previous research focused primarily on the detrimental effects of epibionts, rather than their roles in ecological functioning and ecosystem service provision. We monitored the development and diversity of epibiont assemblages on cultivated sugar kelp (*Saccharina latissima*) at a farm in Cornwall, southwest UK, and compared the effects of different harvesting techniques on epibiont assemblage structure. Increases in epibiont abundance (PERMANOVA, F_4,25_ = 100.56, *p* < 0.001) and diversity (PERMANOVA, F_4,25_ = 27.25, *p* < 0.001) were found on cultivated kelps over and beyond the growing season, reaching an average abundance of >6000 individuals per kelp plant with a taxonomic richness of ~9 phyla per kelp by late summer (August). Assemblages were dominated by crustaceans (mainly amphipods), molluscs (principally bivalves) and bryozoans, which provide important ecological roles, despite reducing crop quality. Partial harvesting techniques maintained, or increased, epibiont abundance and diversity beyond the farming season; however, these kelp plants were significantly fouled and would not be commercially viable in most markets. This paper improves understanding of epibiont assemblage development at European kelp farms, which can inform sustainable, ecosystem-based approaches to aquaculture.

## 1. Introduction

Cultivation of microalgae and macroalgae currently contributes ~20% of total global aquaculture biomass and is rising rapidly at 8% per year [1]. Macroalgal cultivation (i.e., seaweed farming) has recently emerged in Europe, following market expansion and diversification of algal product uses in items ranging from human and animal food to biofuels and bioplastics [1,2,3,4]. Seaweed farming is also increasingly being recognised for its environmental benefits and contributions to ecosystem services [5,6,7,8,9,10,11,12,13]. The vast coastline of northwest Europe is a promising region for the expansion of seaweed cultivation, with cool nutrient-rich waters being favourable for growth of many native and commercially viable species [9,14]. Two major limitations to the expansion of seaweed farming in Europe, however, include a lack of evidence on the potential environmental impacts of farms [9] and biofouling of cultivated biomass by epibionts [15,16,17,18,19].

Biofouling organisms colonise available surfaces in marine ecosystems, including aquaculture infrastructure or as epibionts on farmed seaweed. Epibionts are generally considered pests by farmers as they commonly reduce crop quality and yield, and are estimated to cost the global aquaculture industry USD 1.5–3 billion a year^−1^ [16,20,21,22]. Epibionts in seaweed farms, such as endophytic or epiphytic algae, bryozoans, amphipods and hydroids, may consume or degrade cultivated biomass, inhibit photosynthesis and algal growth, encourage grazers, increase disease susceptibility, contaminate commercial products by introducing allergen or toxin risks, damage farm infrastructure and result in loss of crops due to increased drag during storm events [16,19,21,23,24,25,26,27,28,29,30,31]. Previous studies have therefore mostly focused on the detrimental impacts of epibionts on cultivated seaweed (e.g., [17,18,27,28,32]), rather than the potential benefits of farm-associated biodiversity [21].

Epibionts such as bryozoans, bivalves, sponges, tunicates, and other algae may however improve water quality and host-plant health through biofiltration and nutrient addition [33,34,35], as well as mitigating disease risk [36]. In integrated macroalgal–shellfish farms, seaweed epibionts and their biofiltration services can enhance primary production through nutrient addition and improved water clarity and light penetration, thus benefitting commercially farmed shellfish and encouraging them to settle [21,37,38,39,40,41]. Epibionts may also provide food sources for higher trophic levels, such as fish and macroinvertebrates, potentially increasing the habitat value and secondary production of a cultivation site [8,12,22]. It is therefore important to determine seasonal patterns of epibiont colonisation and the degree of association with other fauna inhabiting seaweed farms to understand how farms may influence local ecosystems and wider ecological functioning. A better appreciation of how epibionts may affect or interact with the environment will also facilitate the development of ecosystem approaches to aquaculture (EAA).

Various studies in European waters have recently examined epibiont assemblages associated with cultivated kelp species (i.e., large brown seaweeds belonging to the order Laminariales), and shown that farms support similar or even higher levels of biodiversity compared to wild populations, by providing novel suspended habitats [42,43]. These studies, however, have generally focused on either the holdfast [42] or the blade [17,18,19,32,43,44] separately, rather than the entire biogenic structure. Quantifying epibiont assemblages across the entire host plant is needed to evaluate the potential habitat value of seaweed farming.

Most studies have focused on epibionts present at harvesting time points (e.g., [26]), when successional processes may be incomplete and assemblages are not yet fully established. This also overlooks the fact that biomass is normally completely removed at harvest time at most farms, thus also removing any biogenic habitat provided by the cultivated seaweed. Furthermore, farmers generally harvest seaweed biomass before any significant biofouling occurs, to maintain crop quality. It is therefore uncertain whether cultivation sites are valuable longer-term habitats, and research is warranted on epibiont assemblage development that extends beyond the growth season [12]. One potential solution to extend the habitat value of seaweed farms is to use partial harvesting techniques, whereby the holdfast and lower blade region are left in place to encourage future growth, with only the top part of the blade harvested [28,29]. Partial harvesting can generate additional yields and reduce seeding costs for farmers [28,29]; however, it is not known whether this approach can maintain or even enhance the potential habitat value of farms as a form of EAA.

The aims of this study were to (i) quantify the development and diversity of successional epibiont assemblages on farmed kelp throughout and beyond its cultivation period at a farm in the Southwest of the UK (Cornwall, England), and (ii) determine whether different partial harvesting techniques maintain epibiont diversity beyond the cultivation period, while still generating commercially viable biomass for farmers. This study was conducted at an integrated sugar kelp (*Saccharina latissima*, Linnaeus [45]) and blue mussel (*Mytilus edulis*, Linnaeus 1758) farm in Cornwall, where no previous quantification of farmed kelp-associated biodiversity has been reported. Kelp plants were collected over and beyond the growing season (winter–summer) and harvested using different partial harvesting techniques. This is also one of the first studies to compare differences in epibiont assemblages between holdfasts and blades of the same cultivated kelp plants over the growing season and within regrowth treatments to assess which structure of the plants was most important for habitat provisioning. Our expectation was that epibiont colonisation would develop and intensify over the growing season, coinciding with increased sea temperatures, reductions in wave energy from winter storms, and the presence of planktonic larval stages in the water column [17,27]. We also predicted that epibiont assemblages would differ between components of kelp plants, with higher diversity in the more sheltered and morphologically complex holdfasts, compared to the more exposed blades, which would instead be dominated by mat-forming or burrowing organisms (e.g., bryozoans and amphipods). Finally, we predicted that implementing different harvesting techniques (such as partial blade removal) would maintain or increase habitat value for an extended period of time, as material is left in situ longer, allowing for colonisation and successional processes to occur [28,29].

## 2. Materials and Methods

### 2.1. Study Site and Farm Setup

**Figure 1:**
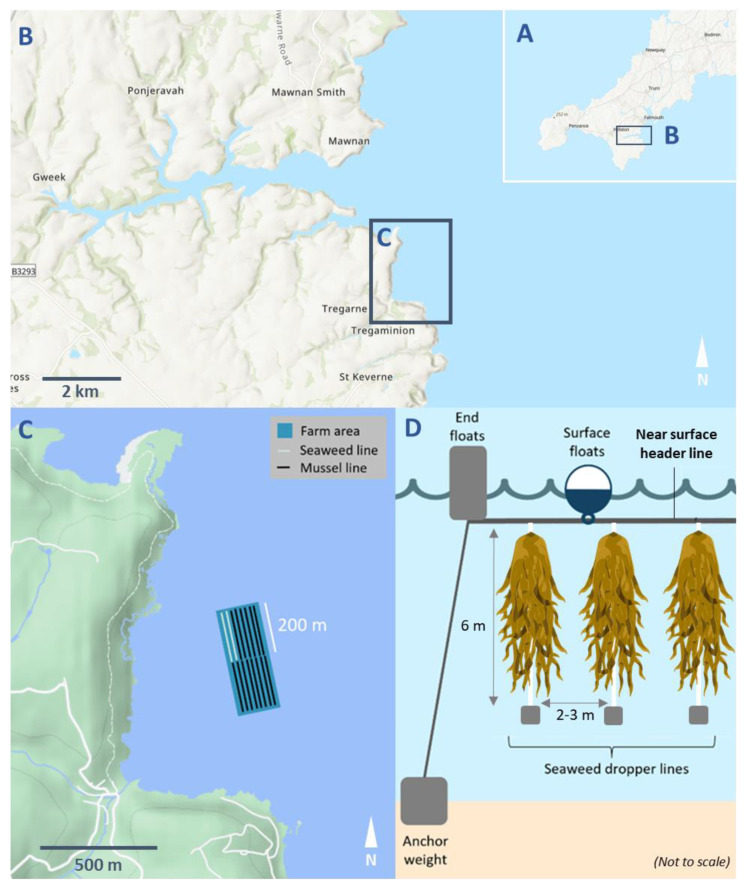
Site map of Porthallow Bay seaweed and mussel farm with (**A**) location in Cornwall, UK; (**B**) position in relation to Helford and Lizard peninsula; (**C**) diagram of the Porthallow farm detailing position of seaweed and mussel lines; (**D**) diagram of the long line system suspending seaweed droppers (not to scale; seaweed graphics are from Biorender (biorender.com)).

Cultivated sugar kelp (*Saccharina latissima*) sporophytes (hereafter ‘plants’) were collected (from April to August 2020 and in May 2021) from an integrated seaweed and blue mussel (*Mytilus edulis*) farm in Porthallow Bay, Cornwall, southwest England, UK (50°04′ N, 5°04′ W) (Figure 1). Porthallow Bay is open to approximately 300 km of wave fetch to the east and southeast, but is sheltered from the predominately south-westerly winds and North Atlantic swells, with the 2 ha integrated farm site situated approximately 500 m from the shore. Porthallow Bay farm site experienced average water temperatures of 13.44 °C (±2.49 SD) (from June 2019 to June 2020), ranging from 9.58 °C (±0.07 SD) in March 2020 to 17.74 °C (±0.14 SD) in July 2019. Average salinity over the same period was 34.78 psu (±0.40 SD), ranging from 32.49 psu (±0.74 SD) in June 2020 to 35.31 psu (±0.06 SD) in September 2019.

The farm uses a longline system with 200 m header lines anchored to the seabed and suspended ~1 m below the surface, supporting either seaweed or mussel growth (Figure 1). The farm is suspended over seabed depths of 4–15 m, with the seabed below consisting of mixed maerl gravel, rocky substrate, and soft sediments.

The farm was seeded in late November 2019 and late October 2020, with *S. latissima* gametophytes attached directly onto 6 m long dropper lines (braided 12 mm AlgaeRope, AtSeaNOVA, Ronse, Belgium) using a binder solution (AtSeaNOVA, Ronse, Belgium). Seeded droppers were then spaced at 2–3 m apart along the header lines suspended at 0.5 m depth from the surface.

### 2.2. Sample Collection

#### 2.2.1. Monthly Sampling

To examine epibiont assemblage development over time, *S. latissima* plants were sampled monthly throughout the main cultivation season (April to June) and beyond (July and August). Each month, 36 kelp plants were collected (by boat) from six independent droppers spread evenly across the two seaweed header lines. From each dropper, three representative plants were randomly selected from both 0–1 m and 3–4 m depth increments, with 216 plants sampled in total. While depth was not a specific factor of interest in the current study, we sampled at the two depth increments to capture any variability along the dropper line, and to capture representative assemblages. Once removed, plants were cut at the base of the stipe and holdfasts and stipes/blades were placed into separate labelled sealable bags. Samples were then frozen at −20 °C within eight hours of sampling and processed at a later date. In May and June 2020, once plants were removed, the density and wet weight biomass of all remaining kelp plants within each depth increment and dropper sampled was quantified, and the total biomass of the whole dropper line was also measured.

#### 2.2.2. Regrowth Sampling

To determine whether biodiversity value may be retained at the farm site beyond the cultivation season (which typically ranges from October–May/June at this site), different harvesting techniques of the *S. latissima* lines were trialled (Figure 1 and Table 1). At harvest (May–June 2020), nine dropper lines were left untouched with all biomass remaining. In October 2020, three of these intact lines were left untouched, three were stripped bare, removing all biomass, and the final three lines were partially harvested above the meristem (i.e., biomass removed by cutting across the blade ~10 cm from the base as per [28]). Regrowth treatments and controls are summarised in Table 1. Regrowth treatment lines remained in situ until the following seaweed harvest in May 2021. The rest of the farm was reseeded as normal in October 2020, as detailed in Section 2.1. In May 2021, *S. latissima* samples from all regrowth treatments and three dropper lines seeded in October 2020 were collected as per Section 2.2.1, whereby three kelps from each depth band (0–1 m and 3–4 m) of each dropper line were sampled.

### 2.3. Sample Processing

All samples were defrosted and rinsed through a 0.5 mm sieve to remove any mobile or loosely attached epibionts (hereafter referred to as mobile epibionts). Samples were then thoroughly examined for any remaining sedentary or sessile individuals and colonial taxa (e.g., bryozoans, ascidians, algae) on both sides of blades or in the interstitial spaces of holdfasts, which were carefully removed where possible. All taxa were sorted into coarse taxonomic groups (Table S1), enumerated, and weighed (wet weight). Colony-forming taxa and mat-forming algae that could not be easily removed were quantified by estimating percentage cover of the blade or holdfast.

Accounting for habitat volume of holdfasts or surface area of blades is important as species richness scales with holdfast and blade size [46]. *S. latissima* samples were measured to attain total plant biomass, maximum blade length and width, holdfast habitable volumes, and biomass of blade and holdfast individually. Blade surface area was calculated approximately using maximum blade width × maximum blade length. Habitable volumes in the holdfasts were calculated using displacement (as described in [47]), by first measuring the volume of water displaced by the holdfast, then subtracting this from the volume of water displaced by the holdfast wrapped in plastic food wrap.

### 2.4. Statistical Analysis

Prior to analysis, the six kelp plants collected from each dropper line were averaged to generate mean values for kelp biometrics (plant biomass (wet weight), blade surface area, holdfast habitat volume) and assemblage metrics (epibiont taxa richness, mobile epibiont abundance, mobile epibiont and algal biomass, and percentage cover of blades by sessile or mat-forming taxa). All analyses were therefore carried out with ‘dropper’ as the spatially independent replicate that encapsulated small-scale (2–3 m) spatial variability between plants. The statistical approaches described below involve univariate and multivariate permutational analyses using the PERMANOVA add-on for Primer v7^®^ software [48,49].

Differences in kelp biometrics and univariate assemblage metrics between months or regrowth treatments for whole kelp plants (i.e., holdfast and blade combined) were examined using one-way permutational analyses of variance (PERMANOVA). Models included “month” or “treatment” as a fixed factor, and permutations (999 under an unrestricted model) were based on Euclidean distances between untransformed data. Pair-wise tests in PERMANOVA were then conducted between months or treatments wherever the main effect was significant (*p* < 0.05).

Variability in multivariate assemblage structure between months or regrowth treatments was examined using PERMANOVA and visualised using metric multidimensional scaling (mMDS) ordination. Multivariate assemblages were examined using the model described above, but with permutations based on separate Bray–Curtis resemblance matrices constructed from the following assemblage metrics: (1) the presence-absence of all taxa; (2) the abundance of mobile taxa; (3) the biomass of mobile taxa and easily detached algae; (4) the percentage cover of sessile taxa. A presence–absence transformation was used for all taxa, as sessile species were not enumerated in the same way as mobile species (i.e., percentage cover of colonies versus abundance of individuals). Fourth root transformation was chosen for abundance and biomass of mobile taxa to down-weight the influence of highly abundant amphipods. Square root transformation was used for percentage coverage of sessile taxa to down-weight any highly abundant taxa. For both the univariate and multivariate metrics, differences in within-treatment variability between levels of factors were also examined using the permutational dispersion (PERMDISP) routine. Where within-treatment dispersion differed between groups, a more conservative *p*-value (*p* < 0.01) was adopted for the main PERMANOVA test for that given response variable [50].

To further examine biodiversity patterns, we then compared assemblages associated with specific kelp plant structures (i.e., holdfast versus stipe/blade) both within and between months or regrowth treatments. Univariate and multivariate analyses were repeated using a two-factor PERMANOVA with “kelp structure” and “month” or “treatment” as fixed factors and permutations (999) conducted under reduced models. Average values presented in text and figures are means ± standard error (SE).

## 3. Results

### 3.1. Epibiont Assemblage Development

**Figure 2:**
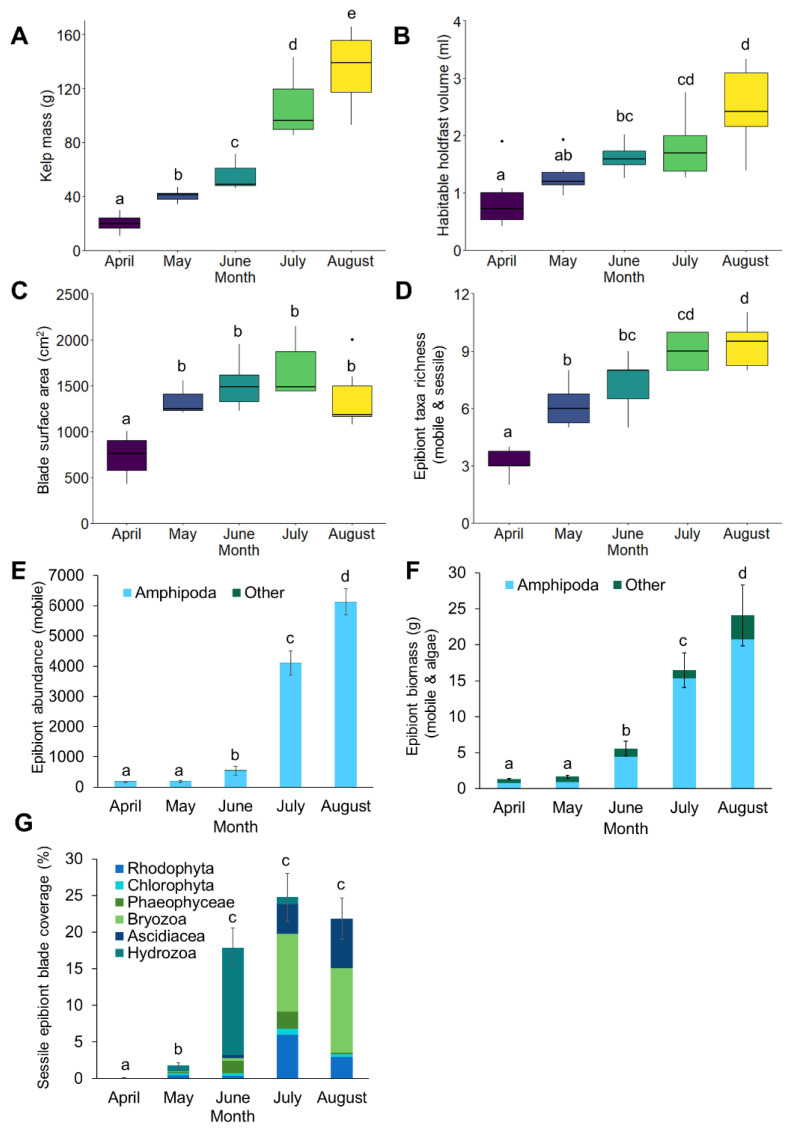
Seasonal monthly averages of (**A**) kelp mass, (**B**) habitable holdfast volume, (**C**) blade surface area, (**D**) taxa richness, (**E**) epibiont abundance for mobile or loosely attached epibionts, (**F**) epibiont biomass for mobile or loosely attached individuals and algae, and (**G**) percentage cover of blade by sessile or mat-forming epibionts. For box and whisker plots (**A**–**D**), the box represents the upper and lower quartiles of the data with the horizontal thicker line representing the median; the vertical line represents the greatest and lowest values, excluding outliers (dots). Significant differences between months are denoted with letters. For bar graphs (**E**–**G**), bars represent the mean with error bars of SE.

*S. latissima* plants developed markedly over the cultivation period (Figure 2) from minimum average blade surface area (735.3 ± 92.3 cm^2^), kelp biomass (20.1 ± 2.7 g), and holdfast habitable volume (0.9 ± 0.2 ml) recorded in April, to maximum blade surface area recorded in July (1664.8 ± 128.9 cm^2^) and maximum kelp biomass (134.6 ± 11.5 g) and holdfast habitable volume (2.5 ± 0.3 ml) recorded in August (Figure 2). Adult kelp density and dropper biomass reached an average of 119.2 kelps m^−1^ (±7.4) weighing 2.6 kg m^−1^ (±0.4) per dropper line in May 2020 and 138.7 kelps m^−1^ (±17.9) weighing 7.7 kg m^−1^ (±0.8) per dropper line in June 2020. Although adult kelp density and dropper biomass were not measured for the rest of the monthly time series, they were observed to consistently increase throughout the summer months.

From the 180 kelp plants processed from April–August 2020, 14 taxa were recorded: 6 from sessile taxa and 8 from mobile taxa (Table S1 in Supplementary, Figure 2). Taxa richness increased significantly over successive months from an average per plant of 3.00 (±0.26) taxa in April, to 9.33 (±0.49) taxa in August (Figure 2, Table 2), although neighbouring months had similar taxa richness values after April (i.e., May–June, June–July, July–August).

A total of 404,158 mobile or loosely attached epibionts were recorded, with the average per plant ranging from 173.60 (±23.01) epibionts with an average total biomass of 1.01 g (±0.11) in April, to 6196.25 (±450.39) epibionts with an average total biomass of 27.53 g (±3.11) in August (Figure 2). There were highly significant increases over months in mobile epibiont abundance and biomass, with pair-wise tests revealing all months differed significantly from one another, except between April and May (Figure 2, Table 2). If these values of epibiont abundance and biomass were scaled to the size of the whole farm (2 ha), we estimate that it would support >16,000,000 epibionts weighing ~100 kg by May and >54,000,000 epibionts weighing ~450 kg by June. If the farm was left unharvested, we estimate it would have supported >618,000,000 epibionts (primarily amphipod crustaceans), weighing ~2750 kg by August (based on assumptions that kelp density per dropper would stay the same as June), in turn, providing a substantial food source for fish species.

Across the growth season, the dominant mobile taxa in terms of abundance were arthropods, i.e., amphipod crustaceans (99.82%), predominantly *Jassa falcata* (Montagu, 1808) (Figure 2). Less dominant taxa included bivalve molluscs (0.15%), isopod, mysid, and decapod crustaceans, gastropod molluscs, echinoderms (Ophiuroidea), and annelid worms (Polychaeta and Oligochaeta) (all <0.1% of total mobile epibiont abundance), which increased in number and diversity over the season (Figure 2). Average percentage cover of sessile epibionts also increased over the season from 0.083% (±0.057) in April to 24.56% (±2.43) in July (Figure 2). Pair-wise tests revealed significant differences in blade coverage between April and May, and between April and May and all summer months (June, July, and August); however, there was no difference between summer months, which all ranged between 17.47 and 24.56% of blades covered by sessile epibionts (Figure 2, Table 2). The dominant sessile taxa in terms of blade percentage cover varied from month to month, with only Phaeophyceae and Rhodophyta (brown and red algal epiphytes) present in April (0.056 ± 0.056% and 0.028 ± 0.028%, respectively), Cnidaria (Hydrozoa) dominating in May and June (0.83 ± 0.34% and 14.58 ± 3.21%, respectively), and Bryozoa dominating in July and August (10.64 ± 2.23% and 11.53 ± 3.81%, respectively) (Figure 2). Other Cnidaria (colonial Ascidiacea) also increased in prevalence over the season to a peak of 6.72 ± 1.63% in August (Figure 2).

**Figure 3:**
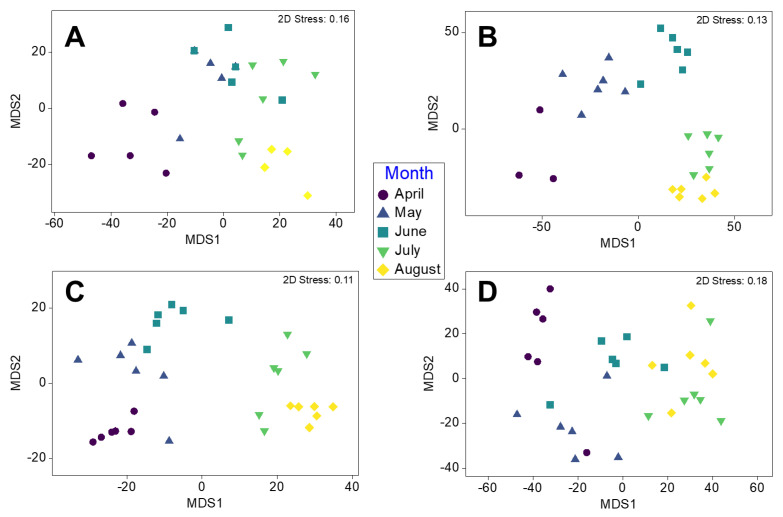
Metric MDS plots depicting multivariate analyses of epibiont assemblages on kelp plants across seasonal months for (**A**) presence–absence of total assemblage including mobile and sessile epibionts, (**B**) percentage coverage of blades by sessile taxa (square root transformed data with dummy variable = 0.4) (**C**) abundance of mobile or loosely attached epibionts (fourth-root transformed), (**D**) biomass for mobile or loosely attached epibionts and algae (fourth-root transformed). All plots are ordinated based on Bray–Curtis similarity matrices of taxa at coarse taxonomic level (i.e., phyla).

Separation in multivariate epibiont assemblage structures (at a coarse taxonomic level, Table S1) between months was evident in the mMDS plots based on presence–absence, mobile epibiont abundance, epibiont biomass and sessile percentage cover of blades, although overlap between neighbouring months was also a common finding (Figure 3). Results of the one-way PERMANOVAs showed significant differences in assemblage composition between months for the presence–absence of all taxonomic groups, mobile abundance, epibiont biomass, and sessile epibiont percentage cover of blades (Figure 3, Table 2). Post hoc pairwise tests showed that for each of these multivariate analyses, all months were significantly different from one another, except for May–June based on total assemblage presence–absence of constituent taxa (Table 2).

#### Differences between Blades and Holdfasts in Monthly Time Series of Epibiont Assemblage Development

**Figure 4:**
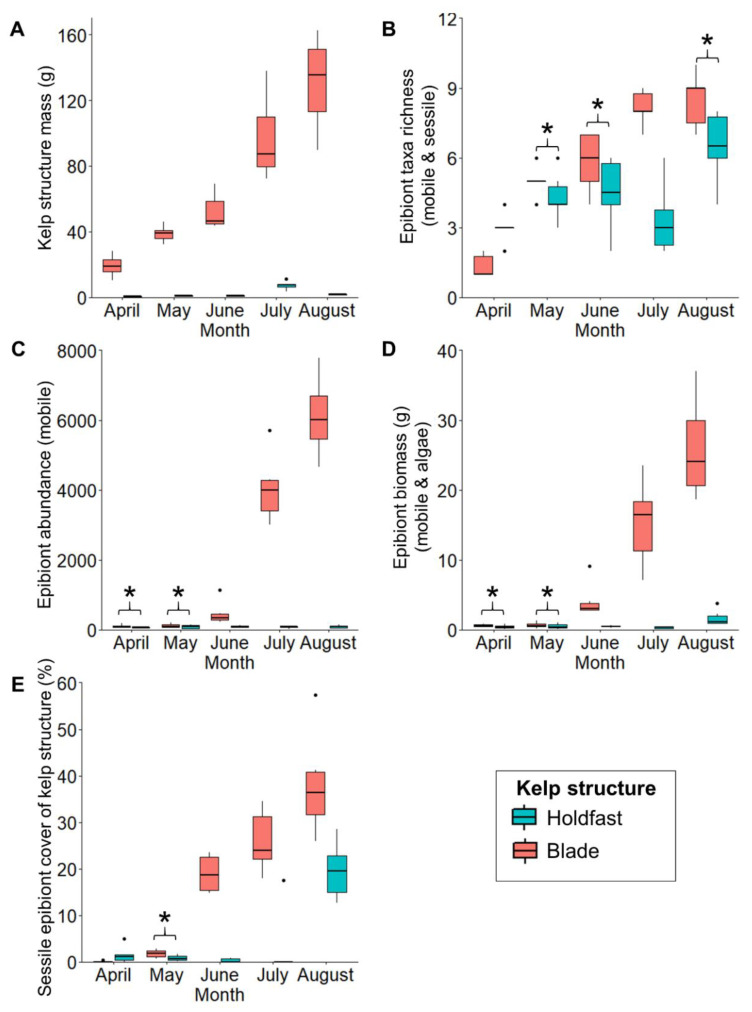
Differences between holdfasts (blue) and blades (pink) between months for (**A**) kelp mass, (**B**) epibiont taxa richness, (**C**) epibiont abundance, (**D**) epibiont biomass, (**E**) percentage cover of kelp structure by sessile epibionts. For box and whisker plots (**A**–**E**), the box represents the upper and lower quartiles of the data with the horizontal thicker line representing the median. The vertical line represents the greatest and lowest values, excluding outliers (dots). Asterisks denote no significant differences between kelp structures.

For all kelp and assemblage metrics, we observed a significant ‘structure × time’ interaction term (Table 3), indicating that differences between kelp structures were not consistent through time. Pair-wise post hoc tests revealed significant and increasing differences in biomass between kelp structures in the same month, with blade mass consistently higher than holdfast mass and thus offering greater biomass (and area) available for epibiont colonisation (Figure 4, Table 3). Regardless, taxa richness increased in both structures of the kelp plant throughout the cultivation period and typically there was no significant difference between kelp structures for most months (Figure 4, Table 3). Taxa richness was however, initially higher in holdfasts in April, and then higher on blades in July (Figure 4). Kelp structures differed increasingly in epibiont abundance, biomass, and percentage cover by sessile taxa through the summer months (June, July, and August); however, initially there were often no differences between kelp structures in April and May (Figure 4, Table 3). Abundance of blade epibionts (predominantly amphipods) increased exponentially with increased blade growth to 6110.69 ± 455.08 individuals per blade in August, while in holdfasts, numbers of epibionts were limited by the smaller habitat area available to 85.55 ± 18.37 individuals per holdfast. Initially sessile percentage coverage was higher on the holdfasts than blades in April, then there was no difference between kelp parts in May; however from June, blades had higher increases in percentage cover, rising to 38.08 ± 4.48% in August compared to 19.58 ± 2.45% on holdfasts (Figure 4).

**Figure 5:**
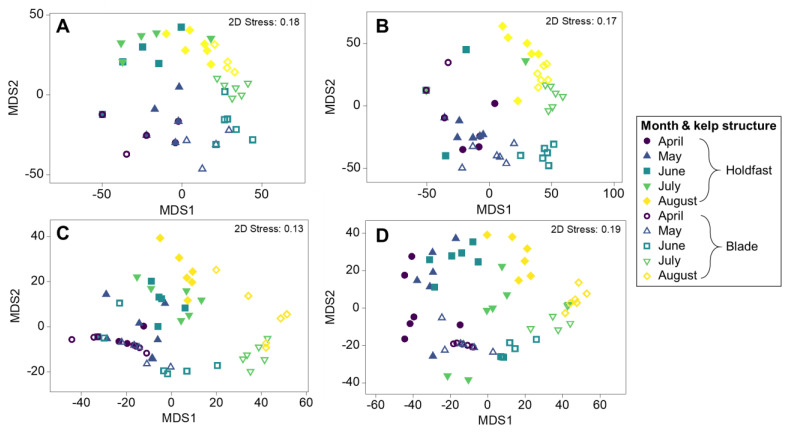
Metric MDS plots depicting multivariate analyses of epibiont assemblages on kelp plant holdfasts and blades across months for (**A**) presence–absence of total assemblage including mobile and sessile epibionts, (**B**) percentage coverage of kelp structure by sessile taxa (square root transformed data with dummy variable = 0.4), (**C**) abundance of mobile or loosely attached epibionts (fourth-root transformed), and (**D**) biomass for mobile or loosely attached epibionts and algae (fourth-root transformed). All plots are ordinated based on Bray–Curtis similarity matrices of taxa at coarse taxonomic level (i.e., phyla, class, order).

Separation in epibiont assemblages (at a coarse taxonomic level) between kelp structures in months was evident in the mMDS plots in terms of phyla presence–absence, mobile epibiont abundance, epibiont biomass, and sessile percentage coverage of kelp structures, although overlap between kelp structures and neighbouring months was also a common finding (Figure 5, Table 3). Post hoc pairwise tests showed that for each of these multivariate analyses, in most months, kelp structures were generally increasingly different from one another (Figure 5, Table 3). There was, however, no difference between kelp structures in terms of total assemblage presence–absence and mobile abundance in April, or percentage coverage of kelp structure by sessile taxa in August (Figure 5, Table 3).

### 3.2. Regrowth Treatments

**Figure 6:**
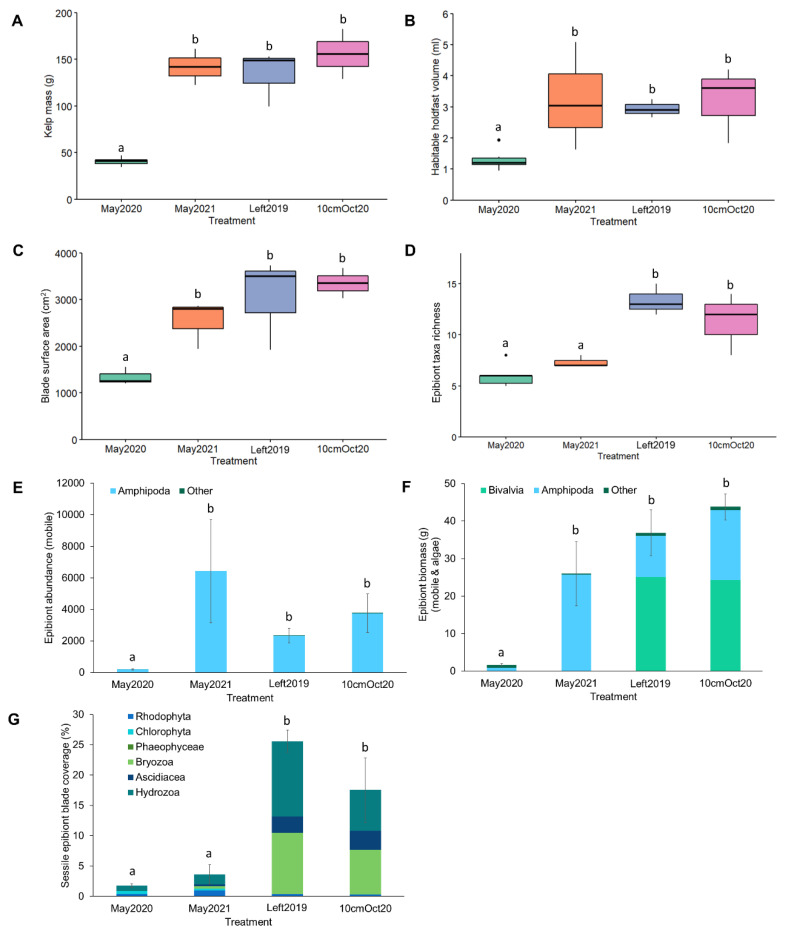
Differences between kelps for control (May2020 and May2021) and regrowth (Left2019 and 10cmOct20) treatments in (**A**) average kelp mass, (**B**) habitable holdfast volume, (**C**) blade surface area, (**D**) taxa richness, (**E**) epibiont abundance for mobile or loosely attached epibionts, (**F**) epibiont biomass for mobile or loosely attached individuals and algae, and (**G**) percentage cover of blade by sessile or mat-forming epibionts. For box and whisker plots (**A**–**D**), the box represents the upper and lower quartiles of the data with the horizontal thicker line representing the median. The vertical line represents the greatest and lowest values, excluding outliers (dots). Significant differences between months are denoted with letters. For bar graphs (**E**–**G**), bars represent the mean with error bars of SE.

Of the three regrowth treatments (Left2019, BareOct20, 10cmOct20), only Left2019 and 10cmOct20 produced any harvestable biomass in May 2021. The BareOct20 treatment did not produce any kelp biomass and was subsequently removed from the statistical analyses. Out of all the control (May2020 and May2021) and regrowth treatments, Left2019 produced the most harvestable average kelp biomass per metre at 3.89 kg m^−1^ (±0.75) in May 2021. It is important to note that the average density of adult kelps in the control treatment May 2021 (25.0 ± 2.11 m^−1^) was approximately five times less than in the previous control treatment May2020 (119.17 ± 6.42 m^−1^); however, the total dropper biomass was greater in May 2021 (1.92 ± 0.35 kg m^−1^) than May2020 (1.71 ± 0.19 kg m^−1^), indicating fewer, but larger kelps on average in May2021 (Figure 6). In terms of habitable area and harvestable biomass, maximum mean blade surface area (4052.25 ± 723.77 cm) and individual kelp weight (187.36 ± 35.31 g) were recorded in the 10cmOct2020 treatment, while greatest mean holdfast habitable volume was recorded in the May2021 control treatment (3.21 ± 0.71 mL) (Figure 6).

In terms of epibiont colonisation, of the 90 kelps processed across the controls and treatments, 16 taxa were recorded: 5 from sessile taxa and algae and 11 from mobile epibionts (Table S1 in Supplementary). In all treatments and controls, the dominant mobile taxa in terms of abundance were amphipod crustaceans (99.34%), followed by bivalve molluscs (0.49%), whereas less dominant taxa included isopod, mysid, and decapod crustaceans, gastropod molluscs, echinoderms, and annelid worms (Polychaeta and Oligochaeta) (all <0.1%), which were present in most samples. Taxa richness differed significantly with treatment (Figure 6), with post hoc tests revealing greater taxa richness in the Left2019 and 10cmOct20 treatments compared to both respective controls (Figure 6, Table 4). A total of 228,514 mobile epibionts were recorded from the regrowth and control treatments, with significantly fewer in May2020 (191.52 ± 37.87 epibionts per kelp with an average mass of 1.14 g ± 0.19) compared to all other treatments (Figure 6). Greatest epibiont abundance was recorded in May2021 (6429.94 ± 3276.71 with an average mass of 25.72 g ± 8.35), and greatest average epibiont mass was recorded in the 10cmOct treatment (43.79 g ± 3.46), predominantly for bivalve molluscs (in particular *M. edulis*) and amphipod crustaceans (Figure 6). Sessile percentage cover of blades was also significantly greater in the treatment types (max: 25.60% ± 1.81 in the Left2019 treatment) compared to the controls (min: 1.75% ± 0.34 in the May2020 control) (Figure 6, Table 4). Across all treatments and controls, the dominant sessile taxa were Hydrozoa (44.63%) and Bryozoa (37.0%) followed by Ascidiacea (12.55%), Rhodophyta (4.33%) and Chlorophyta (1.49%) (Figure 6).

**Figure 7:**
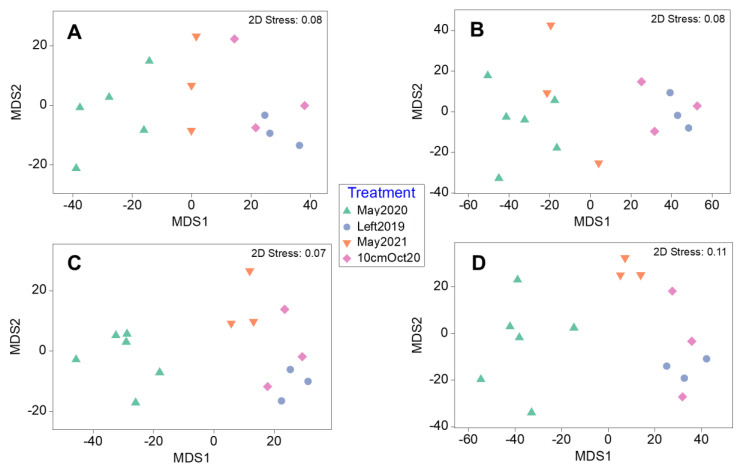
Metric MDS plots depicting multivariate analyses of epibiont assemblages on kelp plants between control and regrowth treatments for (**A**) presence–absence of total assemblage including mobile and sessile epibionts, (**B**) percentage coverage of blades by sessile taxa (square root transformed), (**C**) abundance of mobile or loosely attached epibionts (fourth-root transformed), (**D**) biomass for mobile or loosely attached epibionts and algae (fourth-root transformed). All plots are ordinated based on Bray–Curtis similarity matrices of taxa at coarse taxonomic level (i.e., phyla, class, order).

Separations between regrowth treatments and controls in epibiont assemblages (at a coarse taxonomic level, Table S1) were evident in the mMDS plots in terms of phyla presence–absence, mobile epibiont abundance, epibiont biomass, and sessile percentage coverage of blades, although overlap between treatments was also common (Figure 7). Results of the one-way multivariate PERMANOVAs (999 permutations, unrestricted) showed significant differences in epibiont assemblage composition between treatments for the presence–absence of taxonomic groups, mobile epibiont abundance, epibiont biomass and sessile percentage cover (Figure 7, Table 4). Post hoc pairwise tests revealed that for each of these multivariate analyses, regrowth treatments and the May2021 control were only significantly different from the May2020 control (Table 4), except for there being no significant difference between control treatments in blade percentage cover for sessile taxa.

Total epibiont abundance was significantly greater on the blades than in holdfasts in all controls and treatments, except in the May2020 control, where there was no difference between kelp structures. In control treatments, blades and holdfasts differed significantly in total epibiont biomass, unlike in regrowth treatments where there was no difference between kelp structures, which was most likely due to the attachment of mature *M. edulis* individuals to holdfasts increasing their total biomass (Figure S1, Table S2). Regardless, epibiont taxa richness and percentage cover of the kelp structure did not significantly differ between kelp structures for any treatments or controls (Figure S1, Table S2). According to multivariate analyses, kelp structures typically had significantly different epibiont assemblages within treatments and controls, except for May2020, where holdfasts and blades hosted similar epibiont assemblages in terms of mobile abundance and presence–absence of different taxa (Figure S2, Table S2).

## 4. Discussion

This is the first study to examine the development of epibiont assemblages at a seaweed farm in the UK, a country which, like wider Europe, is experiencing increasing interest in developing the seaweed industry. We found that epibiont abundance, biomass, coverage, and taxonomic richness associated with cultivated *S. latissima* increased throughout, and beyond, the growing season, which highlights the potential for seaweed farms in this region to provide new habitat for a diverse range of taxa including crustaceans, annelid worms, molluscs, bryozoans, ascidians, hydroids, echinoderms, and epiphytic algae. This demonstrates that even small-scale farms, like Porthallow Bay, may support both biodiversity enhancement and the provision of ecosystem services within the IMTA site, such as biofiltration and nutrient regulation [33,34,35] and local fisheries enhancement [8,12,22].

All epibiont taxa present in Porthallow Bay were consistent with those found in previous studies on farmed kelps in regions across Europe [17,19,26,27,28,29,32,42,43,44], and those found on wild kelp populations [42,47,51,52,53]. Increases in epibiont abundance and diversity over the farming season have also been reported at other farms, which tend to follow predictable annual cycles and patterns of ecological succession [19]. Similarly, in this study, the multivariate analyses revealed that taxa do not simply accumulate over time, but undergo successional changes in assemblage composition through the recruitment and replacement of different epibiont phyla as the season progresses. For example, the sessile assemblage composition changed over the season, from hydrozoans being the dominant taxa, to bryozoans, which settle later in May–June [19,32,54]. This pattern, and similar degrees of fouling, were also seen in farm-associated assemblages in west Ireland [19] and Norway [27,32], where bryozoans were also reported as the dominant epibiont by the end of the temporal studies in terms of coverage (up to ~80% of the blade by mid-July in Norway [32]). Sessile epibionts, like bryozoans, are the most commonly reported taxa across European kelp farms, with some studies only focusing on sessile species, and omitting mobile or easily detached taxa from their analyses [18,27,28,32,55]. Focusing on sessile and/or sedentary epibionts that cannot easily be detached from the kelp is useful from an industry perspective due to their detrimental effects on kelp quality; however, this approach does not allow for the total biodiversity and habitat value of farms to be quantified or valued. Furthermore, if this approach was adopted at Porthallow, it would omit amphipods, which dominate mobile epibiont assemblages in terms of both high abundance and biomass and are considered to be the main pests by farmers in this area (personal communication).

Amphipods, such as *J. falcata* (predominating on kelp at the Porthallow site), are small suspension-feeding and tube-building peracarid crustaceans, which can occur in dense aggregations as females brood their offspring, which then recruit to the immediate vicinity [56]. The presence of amphipods and other epibionts, such as bivalves, on farms introduces a shellfish allergen risk into macroalgal food products, which reduces their marketability and increases risks to human health as well as causing kelp breakage [31]. Nevertheless, amphipods, like other mobile epibionts, form important food sources for many fish species, so their presence at farms potentially increases the habitat value and secondary production of a farm site through the creation of rich feeding grounds for fish species [57]. Amphipods, such as *J. falcata* have widespread distributions and have been reported at several other European farms (e.g., [17,19,32,42]), although not in such high abundances as reported here. Amphipods can tolerate a wide range of wave exposures and salinities [58], but population density typically increases with greater wave exposure and turbidity [59]. Furthermore, amphipods like *J. falcata* reach maturity and fecundity earlier in warmer temperatures, with peak reproduction occurring between 10 and 14 °C [60,61]. Porthallow is at a lower latitude compared to most other European seaweed cultivation sites reported upon previously, so the proliferation of amphipods may be due to the warmer water temperatures experienced earlier in the season causing earlier settlement times. Although Porthallow is sheltered from prevailing winds, it is an open bay and experiences a relatively high degree of wave exposure and tidal mixing, compared to other more sheltered seaweed farms (such as those in fjords and lochs), which may also be favourable for amphipods, as well as bryozoans [17,18].

Environmental conditions including temperature, salinity, and exposure are key drivers in epibiont settlement at farms, and explain variation in assemblages across farms in Europe, at local scales, and inter-annually at the same sites [17,18,28,32]. Biotic factors are also important for determining epibiont assemblages at farms, including local larval pools, the species of kelp cultivated and the seeding techniques used [19]. For instance, differences in kelp density and growth in Porthallow Bay between May 2020 and May 2021 control seedings were evident despite using the same seeding method, as significantly higher kelp plant densities were recorded in May 2020 than in May 2021, while overall kelp biomass values were higher in May 2021. Differences in kelp growth between these control treatments were likely due to stormy weather in the first few weeks of deployment of the May 2021 kelps, which likely impacted the epibiont assemblages. To control for inter-annual differences in environmental and biotic conditions in future, more annual cultivation cycles and studies of epibiont colonisation and environmental monitoring should be conducted.

In this study, unlike most previous studies reported on in Europe, the Porthallow seaweed farm is integrated with a *M. edulis* farm, which could also influence which epibionts settle on the farmed seaweed. *S. latissima* grown on the Swedish west coast with *M. edulis* had higher kelp yields in terms of both blade length and biomass and less than half the epiphyte coverage compared to those grown in monoculture in the same region; however, that study did not quantify mobile epibionts [55]. Similarly, in tank cultivation systems, the addition of *M. edulis* and Pacific oysters (*Magallana gigas*, Thunberg, 1793) significantly reduced epiphyte fouling on *S. latissima* blades by ~50% through bivalve filter feeding [62]. Given the rise in integrated multi-trophic aquaculture (IMTA) systems [63,64], future research needs to assess how epibiont assemblages vary for different co-cultured species, to accurately assess the habitat value of the whole farm site [8].

Most previous studies on epibiont colonisation in seaweed farms in Europe have chosen to either focus on the holdfast [42] or the blade [17,18,19,32,43,44] assemblages separately, with disproportionately more research focusing on blade assemblages, due to their commercial value. In this study, we assessed the assemblage of epibionts on whole farmed kelp and compared the assemblages between holdfasts and blades. We found that although blades supported a much higher abundance of epibionts (predominantly amphipods), taxa richness remained similar between holdfasts and blades over the growing season, despite the much smaller size of holdfasts. Holdfast assemblages are generally considered to be the most speciose component of kelp communities due to the complex three-dimensional structure formed by their haptera, which offer a sheltered environment for epibionts that accumulates sediments and organic matter [42,56,65,66,67]. Holdfasts are also slower growing than blades and are less prone to breakage, allowing assemblages to develop over longer time periods [42]. Blades, on the other hand, tend to be relatively smooth, two-dimensional, and more exposed and prone to breakage, providing a less favourable microhabitat for most epibionts [42]. Previous studies on wild kelps in Norway have also found distinct assemblages in holdfasts and blades of the same plant [65]. The differences in epibiont assemblages found between holdfasts and blades in this study highlight the importance of sampling the whole kelp plant to fully assess its habitat value. Understanding the habitat provided by different kelp plant structures is also important for assessments on extending the habitat value of a cultivation site through partial harvesting approaches, whereby blades are mostly removed and holdfasts are left in situ.

The habitat value of seaweed farms is often believed to be temporary, due to the biomass typically being completely removed at harvest before biodiversity can reach a successional peak [12] compared to wild kelps that can live for many years (e.g., wild *S. latissima* is perennial and can persist for 2–4 years [68]). Indeed, in Porthallow Bay, farmers generally aim to harvest the crop in April–June, before amphipod and bryozoan populations in particular have proliferated (personal communication). In this study, harvesting occurred in May, before kelp biomass and epibiont abundance, biomass, and richness had reached a peak, so the habitat currently provided by this seaweed farm was temporary and limited to a few months in duration.

Kelps that were left unharvested or partially harvested in Porthallow continued to grow and provide habitat for epibionts, with increased taxa richness. Unlike in other partial harvesting studies reported in the Faroe and Shetland Islands [28,29], however, the Porthallow kelp biomass was too fouled for farmers to sell profitably for most food markets (personal communication). This may be because biomass had been left in situ for a whole year at Porthallow, compared to only a few months in the studies conducted around the Faroe and Shetland Islands; so shorter regrowth times or an earlier partial harvest (e.g., in May rather than October) would be recommended if farmers wanted to harvest marketable biomass [28,29]. The use of partial-harvesting techniques might usefully be trialled as a way to maintain habitat provisioning by seaweed farms and reduce seeding costs for farmers [28,29] and in turn support an EAA. Future studies might also investigate IMTA systems as a method to help maintain habitat provisioning of cultivation sites, as different species are harvested at different times of the year and may support different epibiont assemblages [8]. Assessments of epibiont colonisation on farm infrastructure that remains in place beyond the cultivation period, such as anchors, header lines and buoys, are also needed to understand the entire habitat value of a farm site, as these may also offer valuable and more permanent substrates for colonisation [16].

Encouraging farmers to maximise habitat provisioning at seaweed cultivation sites may be challenging due to the detrimental effects of biofouling on their crops and farm infrastructure [16]. If the ecological and economic value of habitat provisioning by seaweed farming and the ecosystem services it supports is quantified using standardised techniques [12], this may help to generate additional income to farmers, which would encourage environmental stewardship and the adoption of an EAA. To achieve this, aquaculture farmers need additional support and incentives, like the recent UK Sustainable Farming Incentive for terrestrial farmers, which will reward sustainable farming practices that support food production and benefit the environment [69], or the Farming Investment Fund, which provides funding for equipment, technology, and infrastructure that improves farm productivity and benefits the environment [70].

## 5. Conclusions

Seaweed farms can provide valuable habitat for a wide range of epibiont species, which readily colonise cultivated kelps such as *S. latissima*. Even for small-scale seaweed production sites, such as the 2 ha IMTA site in Porthallow Bay, epibiont abundance and biodiversity can be considerable and the collective biomass could amount to between 100 kg and >2 tonnes of secondary production per year if left unharvested, contributing to local biodiversity and fisheries enhancement and additional ecosystem services, including biofiltration and nutrient regulation. Habitat provisioning and other associated ecosystem services could be retained throughout the year, through partial harvesting of seaweed biomass or using IMTA systems, for example, growing seaweed alongside other farmed products with different growth periods, such as shellfish. These EAA could be encouraged and incentivised through payments for ecosystem services. Now that the ecological benefits of EAA are being quantified in studies such as that presented here, and elsewhere, the next critical step will be to determine the economic value of ecosystem service provisioning. Ecologists need to work with economists and social scientists to engage aquaculture businesses, regulators, and other marine stakeholders in order to ensure sustainable food production through EAA.

## Figures and Tables

**Table 1:** Outline of treatments and controls used in regrowth trials, with the number of replicates given for each treatment (n).

Treatment/ Control Name	Seeding Date	Number of Replicates (n)	Description
May2020 (control)	November 2019	6	Lines from November 2019 seeding, harvested as usual in May 2020
Left2019 (treatment)	November 2019	3	Lines from November 2019 seeding left in the farm an extra year until May 2021 with no harvesting treatment
BareOct20 (treatment)	November 2019	3	Lines originally seeded in November 2019, left in the farm until October 2020, when they were stripped and left in again until May 2021
10cmOct20 (treatment)	November 2019	3	Lines originally seeded in November 2019, left in the farm until October 2020, when they were cut at 10 cm above their meristems and left in again until May 2021
May2021 (control)	October 2020	3	New lines seeded in October 2020, harvested as usual in May 2021

**Table 2:** Results from PERMANOVA and PERMDISP univariate (Uv) and multivariate (Mv) analysis of monthly epibiont assemblage matrices for whole kelp plants, with transformation and post hoc results between months detailed (degrees of freedom (df) are reported within and between treatments).

			PERMANOVA		PERMDISP		
Response Metric	Transformation	*df*	*p*	*F*	*p*	*F*	Post Hoc Significant Differences between Months
(Uv) Taxa richness	N/A	4, 25	0.001	27.245	0.359	1.3115	All different except May–June, June–July, July–August
(Uv) Mobile epibiont abundance	N/A	4, 25	0.001	100.56	0.049	5.1546	All different except April–May
(Uv) Mobile and algal epibiont biomass	N/A	4, 25	0.001	39.471	0.003	8.5812	All different except April–May
(Uv) Sessile epibiont coverage of blade (%)	N/A	4, 25	0.001	24.787	0.011	5.8651	All different except June–July, June–August, July–August
(Uv) Kelp biomass	N/A	4, 25	0.001	87.757	0.014	6.2136	All different
(Uv) Holdfast habitable volume	N/A	4, 25	0.001	7.947	0.42	1.5967	All different except April–May, May–June, June–July, July–August
(Uv) Blade surface area	N/A	4, 25	0.001	10.028	0.417	1.7751	All different except May–June, May–July, May–August, June–July, June–August, July–August
(Mv) Total assemblage (presence–absence)	Presence–absence	4, 25	0.001	14.243	0.265	1.3878	All different except May–June
(Mv) Mobile epibiont abundance	Fourth root	4, 25	0.001	37.333	0.067	2.7666	All different
(Mv) Mobile and algal epibiont biomass	Fourth root	4, 25	0.001	10.093	0.813	0.44907	All different
(Mv) Sessile epibiont coverage of blade (%)	Square root and dummy variable added = 0.4	4, 25	0.001	28.871	0.712	0.6851	All different

**Table 3:** Results from PERMANOVA and PERMDISP univariate (Uv) and multivariate (Mv) analysis of monthly epibiont assemblage matrices for kelp plants split into holdfast and blade structures, with transformation and post hoc results between months and kelp structures detailed.

				PERMANOVA		PERMDISP		
Response Metric	Transformation	Factors	*df*	*p*	*F*	*p*	*F*	Post Hoc Significance Differences between Kelp Structures within Months
(Uv) Taxa richness	N/A	Month	4	0.001	34.987	0.001	8.8306	N/A
		Kelp structure	1	0.001	24.013	0.004	7.8454	N/A
		Month × kelp structure	4	0.001	12.909	N/A	N/A	No difference except in April and July
(Uv) Mobile epibiont abundance	N/A	Month	4	0.001	99.755	0.001	108.52	N/A
		Kelp structure	1	0.001	28414	0.001	145.88	N/A
		Month × kelp structure	4	0.001	99.389	N/A	N/A	All different except in April and May
(Uv) Mobile and algal epibiont biomass	N/A	Month	4	0.001	42.098	0.001	40.775	N/A
		Kelp structure	1	0.001	118.78	0.001	75.951	N/A
		Month × kelp structure	4	0.001	36.051	N/A	N/A	All different except in April and May
(Uv) Sessile epibiont coverage (%)	N/A	Month	4	0.001	58.195	0.001	16.936	N/A
		Kelp structure	1	0.001	78.339	0.005	13.538	N/A
		Month × kelp structure	4	0.001	13.978	N/A	N/A	All different except in May
(Uv) Kelp structure biomass	N/A	Month	4	0.001	393.84	0.001	39.665	N/A
		Kelp structure	1	0.001	40.696	0.001	65.251	N/A
		Month × kelp structure	4	0.001	36.059	N/A	N/A	All different
(Mv) Total assemblage (presence-absence)	Presence–absence	Month	4	0.001	22.981	0.001	12.607	N/A
		Kelp structure	1	0.001	37.167	0.708	0.708	N/A
		Month × kelp structure	4	0.001	9.2638	N/A	N/A	All different except in April
(Mv) Mobile epibiont abundance	Fourth root	Month	4	0.001	23.512	0.001	14.907	N/A
		Kelp structure	1	0.001	27.054	0.001	31.268	N/A
		Month × kelp structure	4	0.001	6.745	N/A	N/A	All different except in April
(Mv) Mobile and algal epibiont biomass	Fourth root	Month	4	0.001	22.904	0.256	1.7008	N/A
		Kelp structure	1	0.001	48.941	0.105	3.0193	N/A
		Month × kelp structure	4	0.001	8.0059	N/A	N/A	All different
(Mv) Sessile epibiont coverage (%)	Square root dummy variable added = 0.4	Month	4	0.001	19.025	0.001	22.106	N/A
		Kelp structure	1	0.001	22.903	0.255	0.84876	N/A
		Month × kelp structure	4	0.001	10.915	N/A	N/A	All different except in August

**Table 4:** Results from PERMANOVA and PERMDISP univariate (Uv) and multivariate (Mv) analysis of different regrowth and control treatments’ epibiont assemblage matrices for whole kelp plants, with transformation and post hoc results between treatments detailed.

			PERMANOVA		PERMDISP		
Response Metric	Transformation	*df*	*p*	*F*	*p*	*F*	Post Hoc Significant Differences between Treatments
(Uv) Taxa richness	N/A	3, 11	0.001	14.401	0.146	2.9333	May2020 and May2021 different from Left2019 and 10cmOct2020
(Uv) Mobile epibiont abundance	N/A	3, 11	0.024	4.0763	0.002	13.744	May2020 different from other treatments
(Uv) Mobile and algal epibiont biomass	N/A	3, 11	0.002	23.752	0.013	6.9953	May2020 different from other treatments
(Uv) Sessile epibiont coverage of the blade (%)	N/A	3, 11	0.001	25.198	0.003	11.83	May2020 and May2021 different from Left2019 and 10cmOct2020
(Uv) Kelp biomass	N/A	3, 11	0.01	16.45	0.089	3.6096	May2020 different from other treatments
(Uv) Holdfast habitable volume	N/A	3, 11	0.02	4.5757	0.046	4.2501	May2020 different from other treatments
(Uv) Blade surface area	N/A	3, 11	0.006	10.558	0.014	6.9989	May2020 different from other treatments
(Mv) Total assemblage (presence-absence)	Presence–absence	3, 11	0.001	11.632	0.7	0.62771	May2020 different from other treatments
(Mv) Mobile epibiont abundance	Fourth root	3, 11	0.001	19.683	0.762	0.7985	May2020 different from other treatments
(Mv) Mobile and algal epibiont biomass	Fourth root	3, 11	0.001	10.882	0.35	2.0862	May2020 different from other treatments
(Mv) Sessile epibiont coverage of blade (%)	Square root	3, 11	0.001	8.8321	0.445	1.5613	May2020 different from Left2019 and 10cmOct2020

## Supplementary Materials

The following supporting information can be downloaded at: https://www.mdpi.com/article/10.3390/life13010209/s1, Table S1: Taxonomic groups used for epibiont classification; Figure S1: Differences between holdfast (blue) and blades (pink) between regrowth and control treatments for (A) kelp mass, (B) taxa richness, (C) epibiont abundance, (D) epibiont biomass, (E) percentage cover of kelp part by sessile epibionts; Figure S2: Metric MDS plots depicting multivariate analyses of epibiont assemblages on kelp plant holdfasts and blades across regrowth and control treatments for (A) presence–absence of total assemblage including mobile and sessile epibionts, (B) percentage coverage of kelp structure by sessile taxa (square root transformed data), (C) abundance of mobile or loosely attached epibionts (fourth-root transformed), (D) biomass for mobile or loosely attached epibionts and algae (fourth-root transformed); Table S2: Results from PERMANOVA and PERMDISP univariate (Uv) and multivariate (Mv) analysis of different regrowth and control treatments’ epibiont assemblage matrices for kelp plants split into holdfast and blade structures, with transformation and post hoc results between treatments and kelp structures detailed.
