# Supplementary material for: Development and Diversity of Epibiont Assemblages on Cultivated Sugar Kelp (Saccharina latissima) in Relation to Farming Schedules and Harvesting Techniques
Source: Life (Basel). 2023 Jan 11;13(1):209. doi: 10.3390/life13010209 (PMC9865293; doi:10.3390/life13010209)
Supplement: Supplementary file 1 [file life-13-00209-s001.zip › life-2134534-supplementary.pdf]

Supplementary material

**Table S1. Taxonomic groups used for epibiont classification**			
**Taxonomic group & Resolution**	**Sessile/ mobile**	**Method of enumeration**	**Present in monthly/ regrowth data?**
**Rhodophyta (phylum)**	Sessile	Percent cover & Presence/absence	Monthly & Regrowth
**Chlorophyta (phylum)**	Sessile	Percent cover & Presence/absence	Monthly & Regrowth
**Phaeophyceae (phylum)**	Sessile	Percent cover & Presence/absence	Monthly & Regrowth
**Bryozoa (phylum)**	Sessile	Percent cover & Presence/absence	Monthly & Regrowth
**Cordata, Tunicata, Ascidiacea (polyphyletic class)**	Sessile	Percent cover & Presence/absence	Monthly & Regrowth
**Cnidaria, Medusozoa, Hydrozoa (class)**	Sessile	Percent cover & Presence/absence	Monthly & Regrowth
**Mollusca, Bivalvia (class)**	Sessile	Count & Presence/absence	Monthly & Regrowth
**Arthropoda, Malacostraca, Amphipoda (order)**	Mobile	Count & Presence/absence	Monthly & Regrowth
**Arthropoda, Malacostraca Mysida (order)**	Mobile	Count & Presence/absence	Monthly & Regrowth
**Arthropoda, Malacostraca Isopoda (order)**	Mobile	Count & Presence/absence	Monthly & Regrowth
**Mollusca, Gastropoda (class)**	Mobile	Count & Presence/absence	Monthly & Regrowth
**Arthropoda, Thecostraca, Cirripedia (subclass)**	Sessile	Count & Presence/absence	Regrowth
**Annelida, Polychaeta and Oligocheata (Worms) (class)**	Mobile	Count & Presence/absence	Monthly & Regrowth
**Arthropoda, malacostraca, Decapoda (order)**	Mobile	Count & Presence/absence	Monthly & Regrowth
**Arthropoda, Pycnogonida, Pantopoda (order)**	Mobile	Count & Presence/absence	Regrowth
**Cnidaria, Hexacorallia, Actiniaria (order)**	Mobile	Count & Presence/absence	Regrowth
**Echinodermata (Phylum)**	Mobile	Count & Presence/absence	Monthly & Regrowth

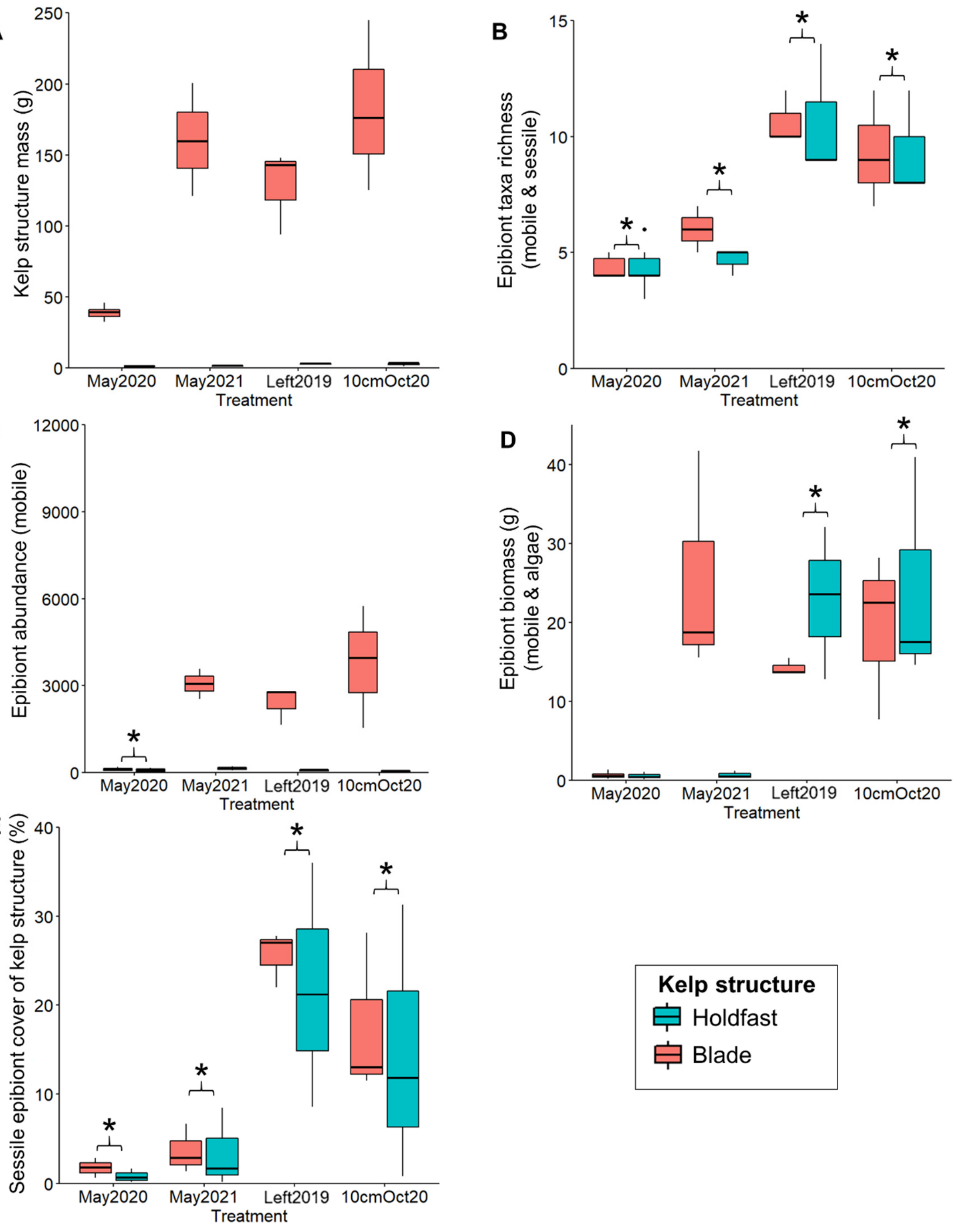

Figure S1. Differences between holdfast (blue) and blades (pink) between regrowth and control treatments for A) kelp mass, B) taxa richness, C) Epibiont abundance, D) Epibiont biomass, E) percent cover of kelp part by sessile epibionts. For box and whisker plots A-E, the box represents the upper and lower quartiles of the data with the horizontal thicker line representing the median, the vertical line represents the greatest and lowest values, excluding outliers (dots). Asterisks denote no significance between kelp structures.

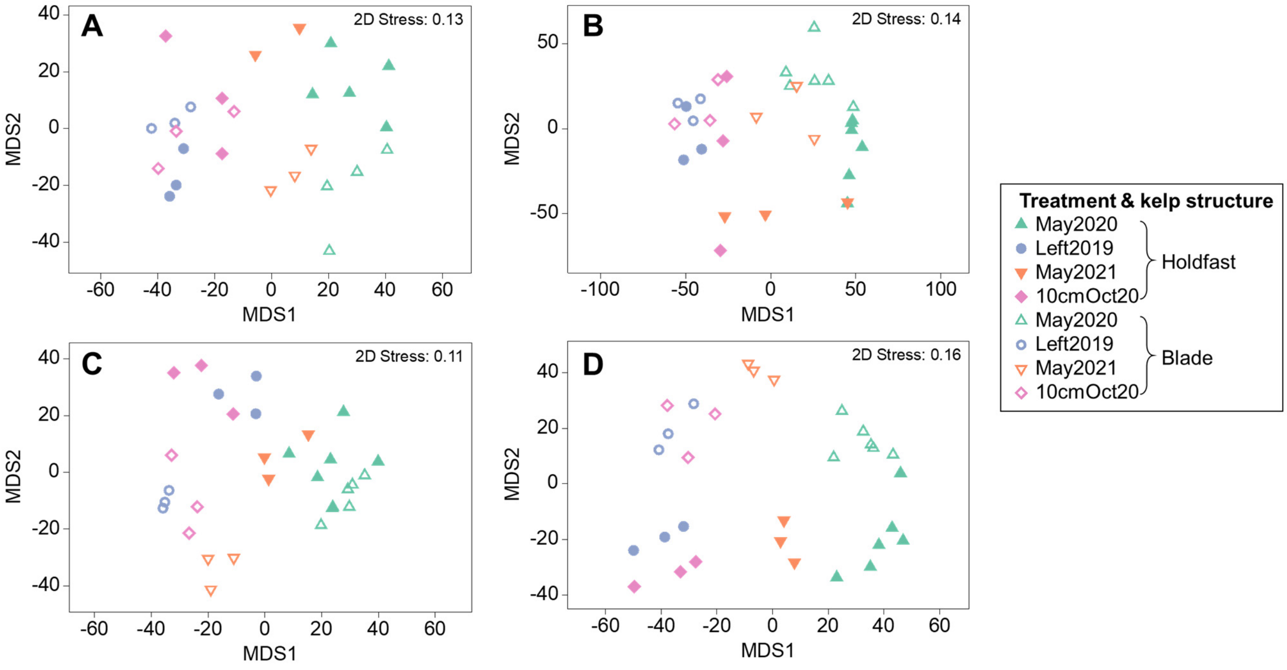

Figure S2. Metric MDS plots depicting multivariate analyses of epibiont assemblages on kelp plant holdfasts and blades across regrowth and control treatments for A) presence-absence of total assemblage including mobile and sessile epibionts, B) percent coverage of kelp structure by sessile taxa (square root transformed data) C) abundance of mobile or loosely attached epibionts (fourth-root transformed), D) biomass for mobile or loosely attached epibionts and algae (fourth-root transformed). All plots are ordinated based on Bray-Curtis similarity matrices of taxa at coarse taxonomic level (i.e. Phyla, Class, Order).

Table S2. Results from PERMANOVA and PERMDISP univariate (Uv) and multivariate (Mv) analysis of different regrowth and control treatments' epibiont assemblage matrices for kelp plants split into holdfast and blade structures, with transformation and post-hoc results between treatments and kelp structures detailed.

				PERMANOVA		PERMDISP		
**Response**	**Transformation**	***df***	**Factors**	**P**	***F***	**P**	***F***	**Post hoc significant differences between kelp structures within treatment**
**(Uv) Taxa richness**	N/A	3	Treatment	0.001	29.333	0.055	3.6725	N/A
		1	Kelp structure	1.4165	1.4165	0.89	0.043146	N/A
		3	Treatment × kelp structure	0.2892	0.2892	N/A	N/A	No differences
**(Uv) Mobile epibiont abundance**	N/A	3	Treatment	0.012	4.1894	0.018	6.4251	N/A
		1	Kelp structure	0.001	19.234	0.001	11.627	N/A
		3	Treatment × kelp structure	0.014	4.08	N/A	N/A	All different except in May2020
**(Uv) Mobile and algal epibiont biomass**	N/A	3	Treatment	0.001	14.125	0.005	8.1788	N/A
		1	Kelp structure	0.334	0.97453	0.671	0.26665	N/A
		3	Treatment × kelp structure	0.004	6.0468	N/A	N/A	Different in May2020 & May2021
**(Uv) Sessile epibiont coverage (%)**	N/A	3	Treatment	0.002	16.642	0.005	8.5603	N/A
		1	Kelp structure	0.491	0.50747	0.935	0.010252	N/A
		3	Treatment × kelp structure	0.958	0.088275	N/A	N/A	No differences
**(Uv) Kelp structure biomass**	N/A	3	Treatment	0.001	16.623	0.002	18.965	N/A
		1	Kelp structure	0.001	193.77	0.001	46.898	N/A
		3	Treatment × kelp structure	0.001	16.047	N/A	N/A	All different
**(Mv) Total assemblage (presence-absence)**	Presence-absence	3	Treatment	0.001	22.229	0.273	1.4997	N/A
		1	Kelp structure	0.001	12.113	0.792	0.06605	N/A
		3	Treatment × kelp structure	0.009	3.2409	N/A	N/A	All different except in May2020
**(Mv) Mobile epibiont abundance**	Fourth root	3	Treatment	0.001	24.542	0.001	8.9823	N/A
		1	Kelp structure	0.001	33.36	0.055	3.9423	N/A
		3	Treatment × kelp structure	0.001	5.6551	N/A	N/A	All different except in May2020
**(Mv) Mobile and algal epibiont biomass**	Fourth root	3	Treatment	0.001	26.591	0.204	1.9181	N/A
		1	Kelp structure	0.001	28.948	0.047	3.863	N/A
		3	Treatment × kelp structure	0.001	7.5708	N/A	N/A	All different
**(Mv) Sessile epibiont coverage (%)**	Square root	3	Treatment	0.001	11.553	0.049	3.9025	N/A
		1	Kelp structure	0.002	6.4712	0.003	11.308	N/A
		3	Treatment × kelp structure	0.012	2.5135	N/A	N/A	All different
